# Mechanism and application of mesenchymal stem cells and their secreting extracellular vesicles in regulating CD4^+^T cells in immune diseases

**DOI:** 10.52601/bpr.2024.240005

**Published:** 2024-12-31

**Authors:** Zehua Lin, Weisong Cai, Yuechen Sun, Baoai Han, Yifan Hu, Zuhong He, Xiong Chen

**Affiliations:** 1 Department of Otolaryngology, Head and Neck Surgery, Zhongnan Hospital of Wuhan University, Wuhan 430071, China; 2 Sleep Medicine Centre, Zhongnan Hospital of Wuhan University, Wuhan 430071, China

**Keywords:** Mesenchymal stem cells, Exosomes, Immune diseases, CD4^+^T cells

## Abstract

Mesenchymal stem cells (MSCs) show significant promise in treating immune diseases due to their ability to differentiate into various cell types and their immunomodulatory properties. However, the mechanisms by which MSCs regulate CD4^+^T cells, essential for immune responses, are not yet fully understood. This study aims to provide a comprehensive overview of how MSCs and their secreted extracellular vesicles (EVs) modulate CD4^+^T cells in immune diseases. We begin by discussing the immunomodulatory properties of MSCs and the factors contributing to their effectiveness. Following this, we explore how MSCs interact with CD4^+^T cells through various pathways, including the secretion of soluble factors, direct cell-cell contact, and EV-mediated communication. A key focus is on the therapeutic potential of MSC-derived EVs, which are rich in bioactive molecules such as proteins, lipids, and nucleic acids. These molecules can regulate the phenotype and function of CD4^+^T cells. The challenges and future perspectives in utilizing MSCs and EVs for immune-disease therapy are also addressed. Overall, this research aims to enhance our understanding of the mechanisms behind MSC-mediated regulation of CD4^+^T cells and provide insights into the potential use of MSCs and EVs as therapeutic tools in immune diseases. In summary, understanding how MSCs and their EVs control CD4^+^T cells can offer valuable perspectives for developing innovative immunotherapeutic approaches. Leveraging the immunomodulatory capacity of MSCs and EVs holds promise for managing immune-related disorders.

## INTRODUCTION

Immune diseases, including autoimmune and allergic conditions, result from disruptions in immune regulation (Cooper and Stroehla 2003; Crowson *et al*. 2011; Rudan *et al*. 2015). The incidence of these diseases has been rising, presenting significant challenges. Prolonged and recurrent episodes can cause irreversible damage to the body and impose substantial economic burdens on individuals and society. Despite advances in drug development, immunomodulation, and biotherapy, these treatments primarily offer symptomatic relief without restoring immune balance or achieving a definitive cure. Therefore, new treatment approaches for immune diseases are urgently needed.

**Figure 1:**
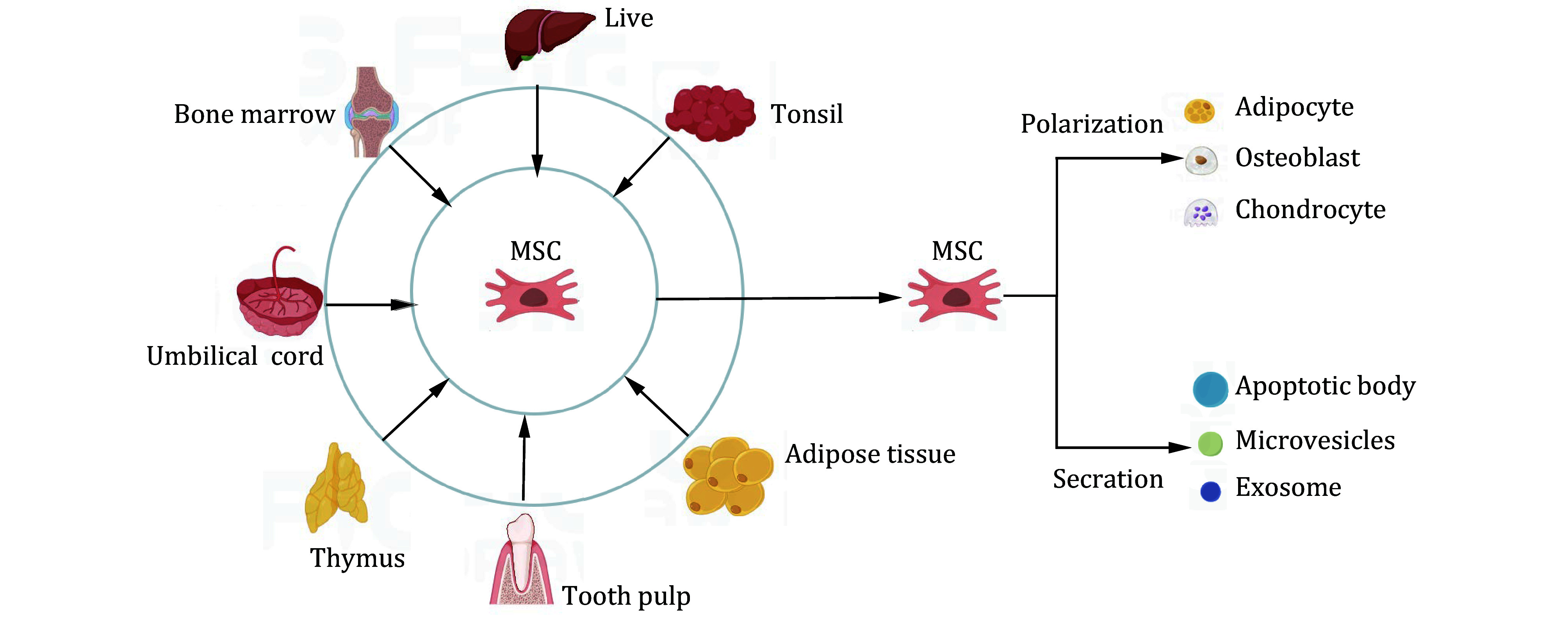
The Origin and differentiation characteristics of MSC. MSCs can be derived from various tissues and possess the capacity to differentiate into cartilage, bone, and fat cells. Additionally, MSCs have the capability to release extracellular vesicles, such as exosomes, microvesicles, and apoptotic vesicles

Recent research has focused on mesenchymal stem cells (MSCs) and their extracellular vesicles (EVs) due to their potential in treating autoimmune diseases (Xu *et al*. 2022). MSCs have also shown promise in addressing disorders of the nervous system (Riazifar *et al*. 2019), cardiovascular system (Bartolucci *et al*. 2017), liver (Yang *et al*. 2023), and kidney (Kou *et al*. 2022). These cells can be derived from various sources, including bone marrow (BM), (Zhang *et al*. 2023) adipose tissue (Yang *et al*. 2022), peripheral blood (Chen *et al*. 2022), synovial tissue (Tarafder *et al*. 2023), and even tonsils (Choi *et al*. 2023) (Fig. 1). Overall, MSCs and their EVs represent a promising area of research, with potential applications across a range of immune and non-immune diseases.

Notably, MSCs have a unique ability to differentiate into mesodermal lineage cells such as adipocytes, osteocytes, and chondrocytes (Hoang *et al*. 2022). Through targeted migration, transplanted MSCs can actively participate in the regeneration and repair of damaged tissues while exerting their immunomodulatory effects via paracrine mechanisms. This offers new hope for patients with immune diseases.

CD4^+^T cells play a crucial role in immune regulation by selectively identifying and binding to specific antigens. They also secrete cytokines that can either enhance or suppress the function of various immune cells (Borsa and Gerlach 2023). The interaction between MSCs and CD4^+^T cells is complex and multifaceted. Studies have shown that MSCs can inhibit the activation and proliferation of CD4^+^T cells, thereby modulating inflammatory responses, autoimmune reactions, and transplant rejection (Akhter *et al*. 2023; Sengun *et al*. 2023). Nevertheless, the relationship between MSCs and CD4^+^T cells is intricate and requires further research to understand the underlying mechanisms fully. Consequently, this article aims to provide a comprehensive overview of the characteristics of MSCs and CD4^+^T cells, along with their regulatory functions in immune-related disorders.

## MESENCHYMAL STEM CELLS

MSCs are adult stem cells capable of differentiating into various cell types from multiple tissues and organs. Currently, the internationally recognized criteria for identifying MSCs are as follows (Dominici *et al*. 2006): (1) Observing colony-like growth in adherent cells; (2) These cells have the capacity to differentiate into adipocytes, osteocytes, and chondrocytes, as depicted in Fig. 1; (3) Expression of the markers CD105, CD90, and CD73, with the absence of CD45, CD34, and CD14. Recent research has increasingly confirmed the immunomodulatory effects of MSCs. *In vitro*, MSCs can inhibit the proliferation and activation of CD4^+^T cells. *In vivo* studies have demonstrated their ability to restore T cell balance in mice with SLE (Cho *et al*. 2023; Hoseinzadeh *et al*. 2023).

## EXTRACELLULAR VESICLES DERIVED FROM MESENCHYMAL STEM CELLS

EVs derived from MSCs play a crucial role in cell-to-cell communication, including exosomes, microvesicles, and apoptotic bodies (Fig. 1). These vesicles, rich in various bioactive molecules, facilitate the transmission of biological information, activate different signaling pathways, and regulate cellular processes (Fang *et al*. 2022; Sullivan *et al*. 2017). Encased in a lipid bilayer, EVs can maintain biological activity for extended periods (Cheruvanky *et al*. 2007). Additionally, EVs exhibit lower immunogenicity compared to direct MSC transplantation, enhancing the treatment's safety profile (Li *et al*. 2021). These unique characteristics have driven extensive research into MSC-EVs as potential alternatives to cell therapy, offering new opportunities for disease management.

## ADAPTIVE IMMUNE RESPONSE INVOLVING CD4^+^T CELLS

Adaptive immunity recognizes pathogens using highly specific antigen receptors produced by lymphocytes through gene rearrangement. CD4^+^T cells bind T cell receptors to MHCII molecules on antigen-presenting cells. When these receptors are co-stimulated with cytokines, CD4^+^T cells proliferate and differentiate into effector or memory T cells, enhancing the adaptive immune response. This process allows CD4^+^T cells to elicit distinct immune responses effectively (Yi *et al*. 2022).

The activation of CD4^+^T cells can be categorized into two ways: TCR signal-dependent activation and TCR signal-independent activation (Tough *et al*. 1997; Whiteside *et al*. 2018). For CD4^+^T cells to become effector T helper cells, they require three distinct signals: (1) TCR engagement with antigen peptide presented by MHC class II molecules (Signal 1). (2) Interaction with costimulatory molecules (Signal 2) to initiate T cell activation. (3) Exposure to various cytokines (Signal 3) (Zhu *et al*. 2010). Once activated, CD4^+^T cells differentiate into various helper T cell subsets, such as Th1, Th2, Th17, Treg, and TFH. These subsets have different functions and are influenced by specific cytokines, playing crucial roles in immune responses.

During intracellular infections, the primary goal of the type 1 immune response is to induce the production of the transcription factor T-bet in CD4^+^T cells. This leads to their differentiation into Th1 cells, mediated by signaling through the IL-12, STAT1, and STAT4 pathways of IFN-γ (Fang *et al*. 2022; Lovett-Racke *et al*. 2004). Th1 cells release type I cytokines, which facilitate the recruitment of CD8^+^T cells and macrophages, enhancing the immune response.

The type 2 immune response is activated in response to extracellular parasites, specific allergens, or weak immunogens. These immunogens promote the expression of GATA3, leading to the differentiation of CD4^+^T cells into IL-4-induced Th2 cells. Th2 cells then release type II inflammatory cytokines, such as IL-4, IL-5, and IL-13. This immune response also recruits mast cells, basophils, and eosinophils to the site of inflammation (Gause *et al*. 2020).

Fungal and extracellular bacterial infections can trigger a type 3 immune response, mediated by the activation of the transcription factor RORγt. This response is initiated by proinflammatory factors such as IL-6, IL-23, IL-1β, or TGF-β. Upon activation, the STAT3 signaling pathway is engaged, leading to the differentiation of T cells into Th17 cells. The type 3 immune response also promotes the recruitment of neutrophils to sites of inflammation (Annunziato *et al*. 2015).

TGF-β1 can induce FOXP^3+^ expression in T cells, which is a key regulatory factor in the differentiation of naive T cells into Treg cells. FOXP^3+^ protein suppresses the expression of intracellular RORγt protein (Fasching *et al*. 2017). The interaction between FOXP^3+^ and RORγt is a significant focus of research in autoimmune disease treatment. Understanding this regulatory mechanism could lead to new therapeutic strategies for managing autoimmune diseases.

## RELATIONSHIP BETWEEN CD4^+^T CELLS AND IMMUNE DISEASES

The abnormal activation and dysfunction of the CD4^+^T cells are key factors in disease progression, leading to the secretion of cytokines and the recruitment of various cells. This process results in persistent abnormal immune responses and inflammatory reactions. An imbalance in helper T cell populations (Th1/Th2, Th17/Treg) is implicated in the pathogenesis of various diseases, including rheumatoid arthritis (RA) (Komatsu *et al*. 2014), inflammatory bowel disease (Szandruk-Bender *et al*. 2022), and SLE (Xiang *et* *al*. 2022).

Abnormally activated CD4^+^T cells can secrete chronic inflammatory factors such as IFN-γ, IL-4, IL-6, and IL-17 (Ogbechi *et al*. 2023; Zou *et al*. 2023). This persistent inflammation damages organs and recruits other immune cells, including B cells, macrophages, and dendritic cells, exacerbating the damage. The loss of immune tolerance causes immune cells to mistakenly attack the body's own tissues, which is another factor contributing to immune diseases (Zhai *et al*. 2023). These mechanisms collectively intensify the immune response and inflammatory reactions, promoting disease progression. Therefore, further research on these mechanisms is essential, and intervention strategies should be developed accordingly.

## REGULATION OF MSCS AND THEIR EXTRACELLULAR VESICLES ON CD4^+^T CELLS IN IMMUNE DISEASES

**Figure 2:**
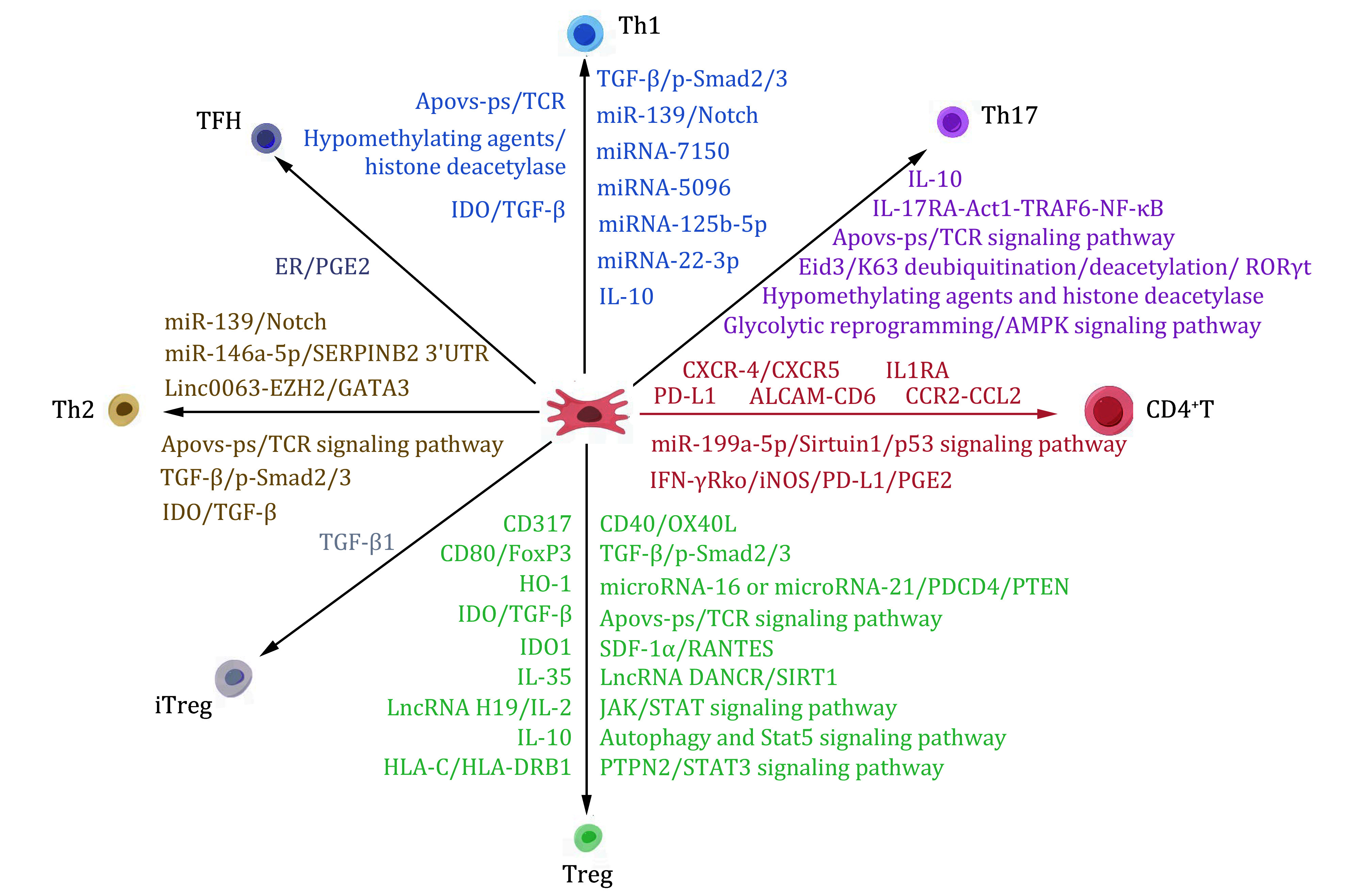
Effects of MSCs and MSC-derived EVs on CD4^+^T in different types of immune diseases. MSCs can secrete or express a variety of proteins, soluble factors and microRNAs, such as IDO1, TGF-β1, miR-139. Substances produced by MSCs have the ability to inhibit and promote the proliferation of CD4^+^T cells, leading to their differentiation into various subpopulations via distinct pathways. These subpopulations include Th1 cells, Th2 cells, Th17 cells, Treg cells, iTreg cells, and TFH cells. The immunomodulatory function of MSCs plays a crucial role in enhancing the regression of immune diseases. For a more comprehensive understanding of the mechanisms underlying the immunomodulatory function of MSCs, please refer to supplementary Table S1

MSCs and their EVs are gaining considerable attention for their ability to regulate the immune system. This section focuses on their regulatory effects on CD4^+^T cells in immune diseases. MSCs and MSC-EVs play significant roles in treating various diseases, including SLE, RA, allergic diseases of the upper respiratory tract, graft-versus-host disease (GVHD), and Autogenous Cerebrospinal Meningitis (ACM). By modulating the number of helper T cells, MSCs and their EVs can effectively reduce the production of inflammatory factors, thereby alleviating the symptoms and inflammatory reactions associated with these diseases. The studies discussed in this section are summarized in supplementary Table S1 and Fig. 2.

### Systemic lupus erythematosus

Systemic lupus erythematosus (SLE) is an autoimmune disease affecting multiple bodily systems. It is characterized by the production and accumulation of autoantibodies, which lead to inflammatory responses and tissue damage (Thanou *et al*. 2021). The development of SLE is closely linked to the breakdown of immune tolerance mediated by CD4^+^T cells. Key pathogenic factors include Th1 and Th17 cells, which secrete cytokines causing organ damage. Additionally, a reduction in Treg cell numbers diminishes the body’s ability to suppress abnormal autoimmune responses, exacerbating the disease.

BMMSCs and BMMSC-EVs have been reported to increase the production of IL-17 and TGF-β, promoting the differentiation of CD4^+^T cells into Th17 cells. This results in reduced proteinuria, single-stranded DNA, complement component 3 (C3), and lymphocyte deposition in MRL/LPR mice, leading to the alleviation of kidney pathology. However, they do not significantly affect the proportions of Treg, Th1, and Th2 cells (Xie *et al*. 2022; Zhou *et al*. 2008).

Another study found that apoptotic vesicles secreted by BMMSCs inhibit Th1, Th2, and Th17 subsets and their cytokines in a dose-dependent manner by binding phosphatidylserine to CD4^+^T cells, thereby alleviating SLE symptoms and pathological manifestations (Wang *et al*. 2023). HuMSCs can promote the differentiation of peripheral blood mononuclear cells in SLE into induced Treg cells by releasing TGF-β1, helping to control SLE (Darlan *et al*. 2021).

The inconsistent regulation of CD4^+^T cell subsets by MSCs in SLE may be attributed to the different sources of MSCs used in various studies. The soluble factors secreted by MSCs from different sources may vary, leading to distinct responses in target cells or organs. BMMSCs can stimulate the proliferation and differentiation of Treg cells and regulate the balance between TFH cells and Treg cells by secreting IL-2 (Chen *et al*. 2021).

HuMSCs can promote the aging of CD4^+^T cells in the spleen by modulating the Sirtuin 1/p53 pathway through the secretion of EVs containing miR-199a-5p, alleviating SLE symptoms in MRL/lpr mice (Cheng *et al*. 2021). MSCs regulate the differentiation of CD4^+^T cells through direct cell contact or paracrine action, exerting immunomodulatory effects mediated by signaling molecules. BMMSC-derived exosomes containing microRNA-16 (miR-16) and microRNA-21 (miR-21) upregulate the expression of anti-inflammatory polarization-related proteins, leading to the generation of anti-inflammatory macrophages and cytokines, such as CD206, B7H4, CD138, Arg-1, and CCL20, while reducing reactive oxygen species production, promoting the differentiation into Treg cells (Sun *et al*. 2022; Zhang *et al*. 2022b).

Recent studies have also highlighted the significance of T follicular helper cells (TFH) in exacerbating SLE, with MSCs being involved in regulating TFH differentiation. BMMSCs or HuMSCs can impede the differentiation of CD4^+^T cells into TFH, ameliorating glomerulonephritis in SLE (Jang *et al*. 2016; Zhang *et al*. 2017). MSCs may suppress the expression of the IL-21 gene or inhibit STAT3 phosphorylation in CD4^+^T cells, hindering their differentiation into TFH and mitigating the disease. Additionally, a decrease in nitric oxide synthase mRNA levels in CD4^+^T cells could serve as a potential mechanism underlying this inhibitory effect (Zhang *et al*. 2017). These studies indicate that MSCs exert their immunosuppressive function by regulating the differentiation and proliferation of CD4^+^T cell subtypes.

### Rheumatoid arthritis

Rheumatoid arthritis (RA) is a systemic autoimmune condition characterized by synovitis, activated cell infiltration, and bone erosion. In RA, IFN-γ secreted by Th1 cells triggers early onset, while IL-17 secreted by Th17 cells aggravates clinical symptoms (Luz-Crawford *et al*. 2015). MSCs have shown promise in modulating immune responses in RA.

Studies have shown that MSCs can increase the number of FoxP^3+^ Treg and IFN-γ^+^ Th1 cells in inguinal lymph nodes in RA mouse models, although they do not affect Th17 cells. The migration of HESC-MSC to these lymph nodes may be linked to the increased expression of indoleamine-2,3-dioxygenase (IDO1) (Gonzalo-Gil *et al*. 2016). Additionally, HuMSC-Exo has been found to reduce joint swelling and synovial hyperplasia in RA mice, decrease proinflammatory cytokines and IgG in serum, and restore the balance between Th17 and Treg cells by down-regulating IL-17 in serum and promoting TGF-β and IL-10 (Fu *et al*. 2022). These findings suggest that exosomes contain key factors for immune regulation. Compared to MSCs alone, MSC-derived EVs have been shown to inhibit T lymphocyte proliferation, reduce IL-17 levels in serum, and promote IL-10 and TGF-β production. HuMSC-EVs also decrease RORγt expression in the spleen, increase FOXP^3+^ expression, and promote T cell differentiation into Treg cells. (Xu *et al*. 2021) Clinical studies confirm that GMSCs inhibit pathogenic Th2 and Th17 cells in RA, significantly improving clinical symptoms (Ghoryani *et al*. 2019; Zeng *et al*. 2022). However, the specific mechanism of MSC immunomodulation on T cells requires further study and clarification.

MSCs migrate to joint lesions under the influence of factors such as stromal cell-derived factor-1α (SDF-1α), activation regulatory factor (RANTES), and sTNFRⅡ. This migration helps protect RA joints from destruction, resist apoptosis, and autophagy induced by TNF-α, and prolong the survival of MSCs *in vivo* (Nam *et al*. 2018; Zhao *et al*. 2021b). GMSC-Exo has been shown to decrease arthritis and bone erosion by blocking the IL-17RA-Act1-TRAF6-NF-κB signaling pathway, inhibiting IL-17A, and promoting IL-10 synthesis (Tian *et al*. 2022). Endoplasmic reticulum stress of HuMSCs releases prostaglandins that inhibit T cell proliferation (Wei *et al*. 2020). BMMSC can induce macrophages to differentiate into M2 type through IL-1 receptor antagonists and regulate CD4^+^T cells (Luz-Crawford *et al*. 2016).

The immunosuppressive ability of MSCs under different culture conditions may differ. Studies indicate that normoxic conditions are more suitable for MSCs to exert their immunomodulatory functions compared to hypoxic or pro-inflammatory cytokine cocktail conditions (Kay *et al*. 2021). BMMSCs can induce the polarization of Th17 cells to Treg cells through the presence of leucine zipper (GILZ) and IFN-γ. However, when GILZ and IFN-γ are knocked out, these MSCs lose their ability to improve joint swelling in rheumatoid arthritis (RA) mouse models (Luz-Crawford *et al*. 2015; Schurgers *et al*. 2010) The immunomodulatory function of BMMSCs may also depend on the expression of inducible nitric oxide, programmed death ligand -1 (PD-L1), and prostaglandin E2 (PGE2) rather than IDO (Schurgers *et al*. 2010). Epigenetic modification is also involved in the regulation of T cells by MSC. Epigenetic modifications play a role in T cell regulation by MSCs. For instance, hypomethylated drugs and histone deacetylase inhibitors can enhance the inhibitory ability of BMMSCs on Th1 and Th17 cells, contributing to the treatment of RA (Kim *et al*. 2018).

Interestingly, MSCs can modulate the immune response and potentially exacerbate diseases. In RA, BMMSCs can engulf apoptotic cells within the bone marrow of RA lesions. This leads to the upregulation of CXCR-4 and CXCR-5 expression in MSCs, promoting their differentiation into osteoblasts. Additionally, these MSCs secrete IL-8, monocyte chemotactic protein-1, and RANTES, which attract CD4^+^T cells to joint lesions, where they adopt a Th17 cell phenotype (Tso *et al*. 2010). This suggests that dysfunctional MSCs may play a pathogenic role in the progression of RA.

Studies have shown that an increased frequency of Tfh cells is associated with higher levels of pathogenic autoantibodies in RA patients, indicating a potential role of Tfh cells in disease development. Allo-MSCs have been found to suppress Tfh cell differentiation in RA patients by producing IDO (Liu *et al*. 2015). Furthermore, using silk fibroin hydrogel containing exosomes derived from PD-L1 olfactory ecto-MSCs can inhibit the PI3K/AKT pathway and effectively impede Tfh cell polarization, offering a potential therapeutic approach for improving RA (Rui *et al*. 2023).

### Upper respiratory allergic diseases (Asthma and AR)

Asthma and Allergic rhinitis (AR) are chronic allergic diseases of the upper respiratory tract caused by excessive or inappropriate immune response to exogenous antigens. Hyper-reactivity and epithelial damage are the main pathological characteristics of AR and asthma, with Th2 cells playing a crucial role. The balance of Th1/Th2 and Th17/Treg cells is essential for maintaining normal immune status. With changes in living environments, the incidence of these allergic diseases is increasing yearly.

### Asthma

Multiple studies have demonstrated that MSCs from various sources can decrease Th2 cells in lung tissue and the thymus, suppress IL-4 levels, and enhance Th1-related cytokine IFN-γ along with the key transcription factor T-bet. They also increase TGF-β1 and PGE2 content in asthma models, leading to reduced eosinophil infiltration and collagen fibrosis in lung tissue, and partially restoring Treg cell numbers (Cruz *et al*. 2015; de Castro *et al*. 2017; Keyhanmanesh *et al*. 2018; Shin *et al*. 2021). BMMSCs and DFSCs achieve this by inhibiting CD4^+^T cell proliferation through the induction of IFN-γ, PGE2, IDO, and TGF-β1 for immune regulation (Genç *et al*. 2018; Goodwin *et* *al*. 2011). External addition of IFN-γ or the use of TGF-β1 neutralizing antibody or TβR1 inhibitor can reverse MSC resistance to airway inflammatory factors and decrease MSC migration into the airway. Notably, blocking PGE2 does not reverse these effects (Aleahmad *et al*. 2021; Dai *et al*. 2019; Gao *et al*. 2014; Nozari *et al*. 2022). Silencing IDO in ADMSCs significantly inhibits Treg cell proliferation, suggesting that inhibiting IDO expression in MSCs could be crucial for immune-related disease (Heidari *et al*. 2021)

Furthermore, increased PD-L1 expression in CD4^+^T cells has been observed with MSC intervention in asthma. Some studies indicate that MSCs may upregulate miR-139 expression in lung tissue, activate the Notch pathway, enhance CXCR4 expression in bronchial epithelial cells, and suppress Th2 cells inflammatory response (Castro *et* *al*. 2020; Wang *et al*. 2021). MSCs can also regulate the CCR-CCL2 axis to inhibit CD4^+^T cell proliferation and reduce lung tissue damage. Additionally, MSCs treated with both IFN-γ and TNF-α have shown efficacy in reducing Th9 cell numbers and alleviating asthma-related allergies compared to untreated MSCs (Nozari *et al*. 2022).

### Allergic rhinitis

MSCs can effectively improve nasal symptoms of AR mice by reducing inflammatory infiltration, lowering OVA IgE expression in serum, inhibiting Th17 cell proliferation, promoting Treg cell generation, and restoring Th17/Treg cell balance (Zou *et al*. 2021). Exosomes containing Linc00632 secreted by MSCs can interact with EZH2 in T cells, inhibit GATA3 expression, reduce T cell differentiation into Th2 cells, and improve AR-related symptoms in mice (Li *et al*. 2022b). MSCs-EXO containing miR-146a-5p can bind to with the 3′-UTR of SERPINB2 in CD4^+^T cells to inhibit T cells to Th2 differentiation, thereby controlling inflammation (Zhou *et al*. 2021a). MSCs expressing ALCAM can combine with the CD4^+^CD25^–^T cell receptor CD6 to inhibit T cell proliferation. Neutralizing ALCAM or CD6 counteracts the inhibitory effect of MSCs on T cell proliferation, and silencing ALCAM in MSC negates their ability to inhibit IFN-γ secretion by T cells, preventing T cell-mediated tissue damage (Cho *et al*. 2023). The morphology of MSCs may explain their immune regulation function. Increased expression of ICAM-1 and VCAM-1 proteins related to cell morphology enhances their ability to inhibit T cell proliferation (Silva-Carvalho *et al*. 2022). Dysfunctional MSCs have a significantly lower capacity to restore T cell balance compared to normal MSCs, which can contribute to the progression of immune diseases by increasing IL-17 inflammatory factors and inducing a high-inflammation environment (Li *et al*. 2023; Zhao *et al*. 2021a).

In summary, while asthma and AR are both airway diseases, the different cytokines involved may play distinct biological roles. Therefore, applying MSCs in the clinical recovery of the immune status in patients with respiratory allergic diseases requires extensive preclinical research.

### Graft-versus-host disease

GVHD is a common immune complication following allo-HCT, where immune cells in the graft attack the recipient's tissues and organs, resulting in a range of clinical symptoms and complications. Currently, treatment involves glucocorticoids combined with immune drugs, but there is no effective treatment for refractory GVHD. The emergence of MSCs offers a potential new therapeutic approach for this disease.

In GVDH, MSCs have been shown to rapidly restore the homeostasis of CD4^+^T cells and positively regulate the expression of related inflammatory cytokines (IL-6, IL-8, IL-17, TNF-α, IFN-γ, G-CSF, GM-CSF, PDGF-BB, FGF-b, and IL-5), compared to standard GVHD treatment. MSC-Exo can also reduce Th1 cells (Petinati *et al*. 2023; Zhang *et al*. 2022a). The exogenous addition of heme oxygenase-1 significantly enhances the inhibitory effect of MSCs on acute exclusion reaction (Li *et al*. 2022a). Apparently, rapamycin has been found to enhance the rejection of MSCs in liver transplantation models and prolong the survival of rats, suggesting that autophagy may play an important role in the immune regulation of MSCs (Zhou *et al*. 2021b). Further research indicates that MSC-CM may regulate the expression of FOXP^3+^ by activating autophagy and the Stat5 signaling pathway, promoting the proliferation of Treg cells and restoring the immune status of patients (Zhang *et al*. 2022a). MSCs overexpressing CD40 can inhibit OX40L, thereby restoring the balance of Treg cells, promoting normal B cell development, and reducing the occurrence of acute rejection in bone marrow transplantation. Conversely, BMMSCs with CD40 knocked down can reduce the number of B cell precursors and promote the occurrence of GVHD (Bassani *et al*. 2021) MSCs overexpressing CD80 and IL-35 or with CD137 knocked down can also play a similar role (Guo *et al*. 2022; Kay *et al*. 2022; Mittal *et al*. 2022). Moreover, the long non-coding RNA (lncRNA) DANCR in BMMSCs-Exo binds to SIRT1 in CD4^+^T cells, leading to SIRT1 ubiquitination and down-regulation, promoting the differentiation of CD4^+^T cells into Treg cells. This reduces the content of serum creatinine and plasma rejection-related factors, establishing immune tolerance in allogeneic kidney transplantation models in mice (Wu *et al*. 2022). Treatment with HuMSC-EVs has been shown to alleviate chronic GVHD (cGVHD) scores and skin fibrosis in cGVHD mice. This therapeutic effect may be attributed to MSC-EVs inhibiting TFH and germinal center B cells interactions, and decreasing the ratio of B cells activating factor to B cells *in vivo*, thereby modulating B cell immune responses (Guo *et al*. 2020).

### Sjogren's syndrome

The Sjogren's Syndrome (SS), often presenting with dry eye syndrome as the first symptom, is an autoimmune disease with limited immunotherapy options. Research indicates that MSCs show promise in improving the prognosis of SS. Specifically, studies have demonstrated that treatment with BMMSCs can promote the differentiation of T cells into Treg and Th2 cells while suppressing Th17 and Tfh responses, thereby alleviating disease symptoms.

BMMSCs, when infused into the body, migrate to inflamed areas in a manner dependent on stromal cell-derived factor 1. Blocking the CXCR4 ligand of this factor diminishes the efficacy of BMMSC therapy (Xu *et al*. 2012). Additionally, BMMSCs have been shown to decrease the populations of Th1, Th17, and Tfh cells in SS mice while increasing the proportion of Treg cells by reducing IL-12 levels in dendritic cells. These findings highlight the significant role of Tfh cells in the immune modulation by MSCs (Shi *et al*. 2018). Further investigations reveal that suppressing PD-L1 in olfactory ecto-mesenchymal stem cell-derived exosomes (OE-MSC-Exos) can effectively impede the progression of experimental Sjogren's Syndrome in mice by inhibiting the differentiation of T cells into Tfh cells (Rui *et al*. 2022). These studies suggest the research value of MSCs and their exosomes in the treatment of SS diseases.

### Autogenous cerebrospinal meningitis

ACM is an autoimmune disease that causes a range of neurological symptoms due to the immune system's T cells and B cells attacking normal brain and spinal cord tissues. MSCs can significantly reduce the number of pathogenic Th1 and Th17 cells in EAE, a model of autoimmune meningitis. This effect is likely related to the inhibition of the co-stimulatory ability of antigen-presenting cells and B cells, thereby reducing the severity of EAE (Li *et al*. 2022c). Compared with MSCs, MSC-EVs are more effective in lowering the clinical scores of EAE mice and improving tissue lesions. MSC-EVs promote the differentiation of CD4^+^T cells into Treg cells, reduce the levels of proinflammatory cytokines IL-17a, TNF-α, and IFN-γ, and increase the levels of the anti-inflammatory cytokine TGF-β. They also elevate IL-4 and IL-10 levels, which help inhibit immune responses and reduce inflammation (Ahmadvand Koohsari *et al*. 2021; Fathollahi *et al*. 2021). Additionally, MSC-EVs treated with EP300 immunosuppressant Eid3 can promote the de-ubiquitination and deacetylation of K63 in Th17 cells, leading to the degradation of the main regulatory factor RORγT protein. This process transforms Th17 cells into EX-Th17 cells, which have low IL-17 production, thereby reducing the number of Th17 cells in the central nervous system. This reduction controls the clinical symptoms of EAE mice, suggesting that MSC-EVs can be used as a novel immunosuppressant (Jung *et al*. 2023).

### Other immune diseases

In autoimmune uveitis, MSCs can also significantly reduce Th17 cells in lymphocytes and increase the number of regulatory B cells, thereby inhibiting the differentiation and activity of interleukins in experimental autoimmune uveitis (EAU) rats (Mu *et al*. 2022). Both *in vivo* and *in vitro* experiments have further confirmed that MSC-EVs overexpressing IL-10 can significantly reduce the clinical and histological scores of EAU mice. They also reduce the proportion of Th1 and Th17 cells in the eye while increasing the number of Treg cells in the spleen and lymph nodes. *In vitro* experiments show that MSC-EVs overexpressing IL-10, when co-cultured with T cells, can increase T cell proliferation and activation, inhibit the differentiation of T cells into Th1 and Th17 cells, and promote their differentiation into Treg cells. This indicates that MSC-EVs containing IL-10 can aggregate in target organs, be internalized by T cells, and play an immunoregulatory role (Li *et al*. 2022d).

In other immune diseases, the molecular mechanisms by which MSCs regulate T cells may involve various pathways, including the JAK/STAT signaling pathway (Kim *et al*. 2022), PTPN2/STAT3 signaling pathway (Gao *et al*. 2022), microRNAs (Gong *et* *al*. 2021). These mechanisms may also depend on active mitochondrial transfer mediated by HLA-C and HLA-DRB1 (Akhter *et al*. 2023; Piekarska *et al*. 2022). Additionally, after metabolic reprogramming of MSC glycolysis, MSCs can significantly improve the prognosis of a delayed-type hypersensitivity mouse model through the AMPK pathway (Contreras-Lopez *et al*. 2021). In summary, MSCs and MSC-EVs offer promising therapeutic potential for autoimmune uveitis and other immune disorders by modulating immune cell activity and reducing inflammation. Further research is necessary to fully elucidate their mechanisms and optimize their therapeutic application.

## SECURITY ISSUES OF MSCS AND MSC-EVS

MSCs and MSC-EVs are at the forefront of stem cell research, with extensive study and application in the field. MSCs have emerged as a promising area of immunotherapy due to their immune regulatory properties, secretion of bioactive factors, and diverse sources. However, the heterogeneity and safety concerns associated with MSCs limit their application. For instance, some studies have found that MSCs can be tumorigenic and may cause vascular embolism, potentially leading to fatal outcomes (Lee *et al*. 2013; Moll *et al*. 2019). It has been suggested that the infusion of highly procoagulant tissue factor TF/CD142 should be the minimum standard for determining the suitability of MSCs for intravascular injection (Moll *et al*. 2022). Thus, effectively evaluating the safety of MSCs remains an unresolved issue. The development of MSC-EVs offers new hope for immunotherapy. Compared to MSCs, MSC-EVs exhibit low immunogenicity, are cell-free, and provide stability. However, controlling the quality of isolated MSC-EVs remains challenging.

In summary, while MSCs and MSC-EVs hold significant promise for immunotherapy, further research is needed to address safety concerns and improve the quality control of MSC-EVs to ensure their effective and safe application in clinical settings.

## SUMMARY AND PROSPECT

MSCs and their derived EVs have great potential for treating immune diseases. By regulating the proliferation and differentiation of CD4^+^T cells, MSCs can restore the body's immune state, offering possible treatment options for personalized and precise therapies. Recent research suggests that Th22 cells play a role in the development of various immune disorders. However, it remains uncertain whether MSCs can mitigate disease progression by modulating Th22 cells, highlighting a potential direction for future research. Despite their promise, controlling the quality and safety of MSCs and MSC-derived EVs remains a significant challenge. Numerous preclinical studies are needed to address these issues and ensure their safe and effective application in clinical settings.

## Conflict of interest

Zehua Lin, Weisong Cai, Yuechen Sun, Baoai Han, Yifan Hu, Zuhong He and Xiong Chen declare that they have no conflict of interest.
